# Supplementary material for: Establishment of the First National Standard for Neutralizing Antibodies against SARS-CoV-2 XBB Variants
Source: Viruses. 2024 Apr 1;16(4):554. doi: 10.3390/v16040554 (PMC11053542; doi:10.3390/v16040554)
Supplement: Supplementary file 1 [file viruses-16-00554-s001.zip › viruses-2942975-supplementary.pdf]

Table S1. Information about donors of three candidate standards (No. 11, 22 and 33).

Candidate standards	Sex	Age	Region	Severity of COVID-19 symptoms
11	Male	51	Jiangxi Province	Severe
	Female	49	Jiangxi Province	Mild
	Male	43	Chongqing	Severe
22	Female	58	Jiangxi Province	Mild
	Male	46	Hebei Province	Mild
	Male	35	Chongqing	Severe
33	Male	24	Henan Province	No

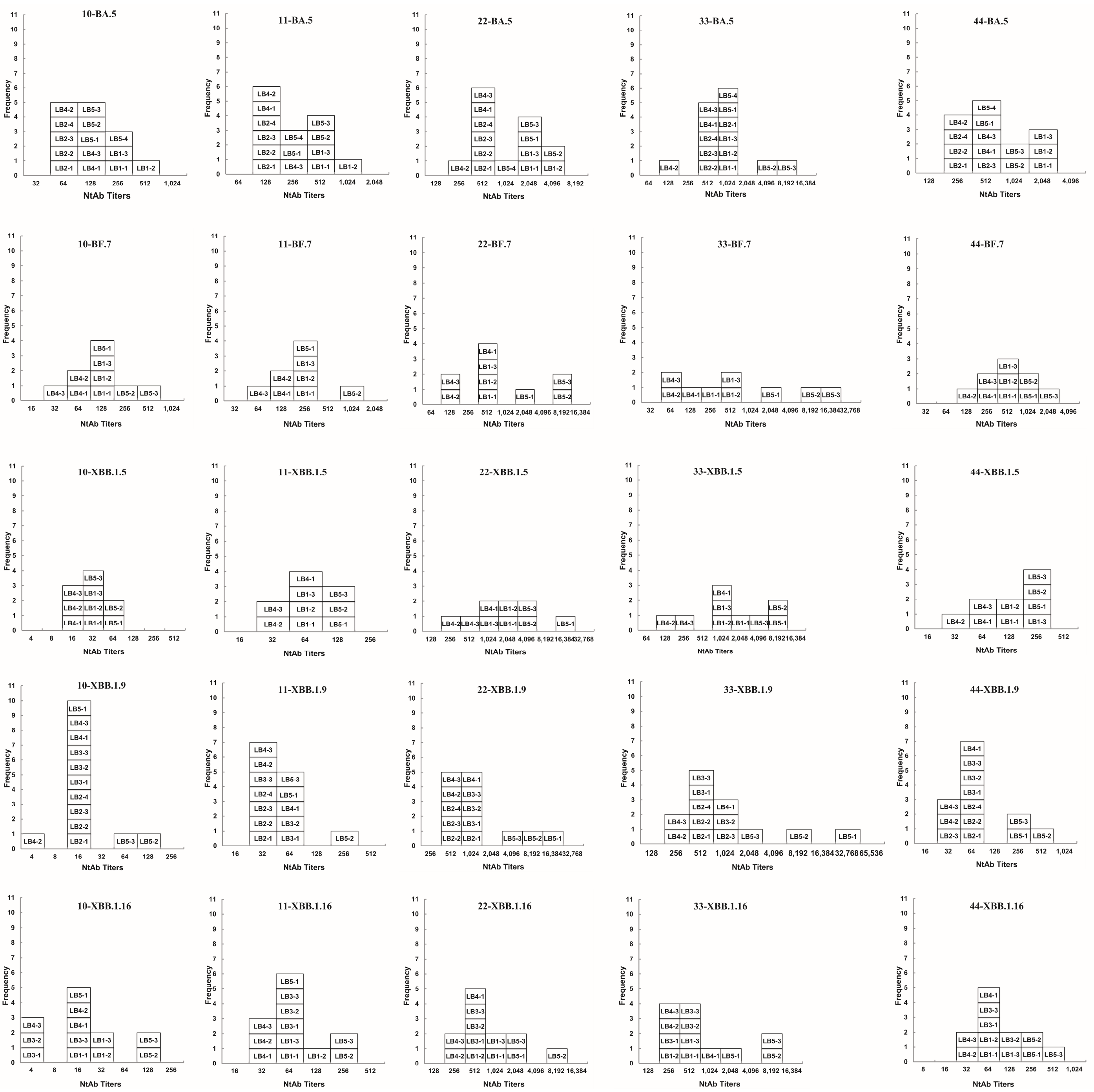

Figure S1. Histograms showing the distribution of the Neut assay endpoint titer (ID50) for four candidate standards (No.11, 22, 33 and 44) across laboratories. Each box represents the endpoint titer for each assay, labelled with the laboratory code number (LB1–5), and followed by one independent assay (1–4), indicating the assays. The Neut results using BA.5, BF.7, XBB.1.5, XBB.1.9, XBB.1.16 variants are shown row by row.

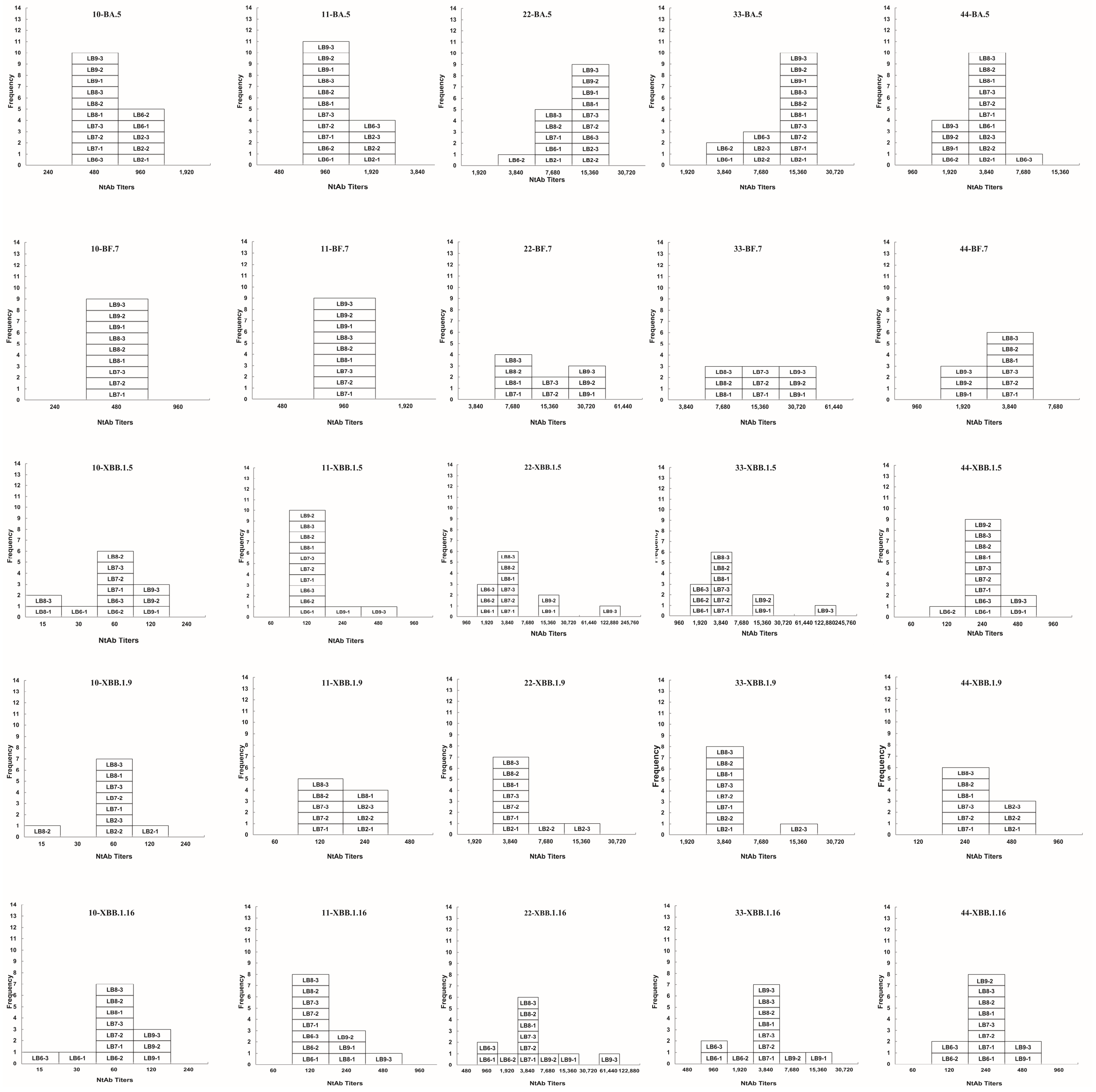

Figure S2. Histograms showing the distribution of the PsN assay endpoint titer (ID50) for four candidate standards (No.11, 22, 33 and 44) across laboratories. Each box represents the endpoint titer for each assay, labelled with the laboratory code number (LB2, 6–9), and followed by one independent assay (1–4), indicating the assays. The PsN results using BA.5, BF.7, XBB.1.5, XBB.1.9, XBB.1.16 variants are shown row by row.

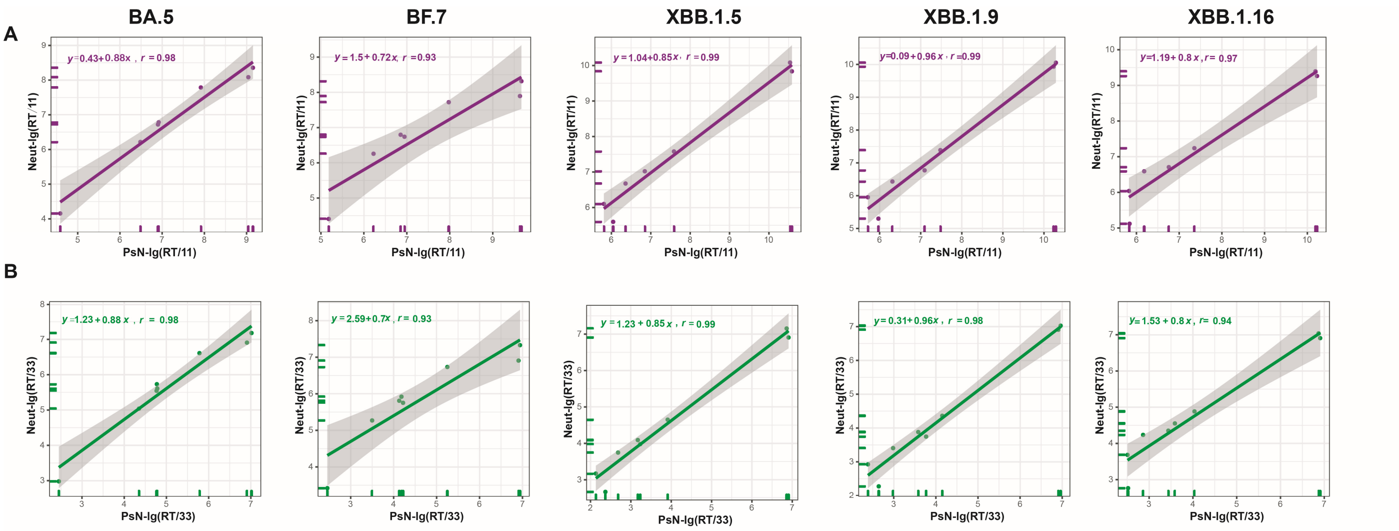

Figure S3. Correlation of RT/11 and RT/33 between the PsN and Neut methods.
